# PARP inhibitors in the treatment of ARID1A mutant ovarian clear cell cancer: PI3K/Akt1-dependent mechanism of synthetic lethality

**DOI:** 10.3389/fonc.2023.1124147

**Published:** 2023-02-22

**Authors:** Vasily A. Yakovlev, Stephanie A. Sullivan, Emma C. Fields, Sarah M. Temkin

**Affiliations:** ^1^ Department of Radiation Oncology, Massey Cancer Center, Virginia Commonwealth University, Richmond, VA, United States; ^2^ Gynecologic Oncology Division, Massey Cancer Center, Virginia Commonwealth University, Richmond, VA, United States

**Keywords:** AKT1, ARID1A, homologous recombination repair, ionizing radiation, non-homologous end joining repair, ovarian clear cell carcinoma, PARP inhibitors, PI3K

## Abstract

**Introduction:**

Poly(ADP-ribose) polymerase (PARP) is a nuclear enzyme involved in the repair of DNA single-strand breaks (SSB). The recent development of poly(ADP-ribose) polymerase inhibitors (PARPi) results from over 45 years of studies. When the activity of PARP1 or PARP2 is compromised, DNA SSB lesions are unresolved and can be converted to DNA double-strand breaks (DSBs) by the cellular transcription mechanisms. ARID1A (also called BAF250a) is an important component of the mammalian Switch/Sucrose Non-Fermentable (SWI/SNF) chromatin-remodeling complex. ARID1A gene demonstrates >50% of mutation rate in ovarian clear-cell carcinomas (OCCC). Mutated or downregulated ARID1A significantly compromises the Homologous Recombination Repair (HRR) of DNA DSB.

**Results:**

The present study demonstrated that downregulated or mutated ARID1A attenuates DNA HRR through stimulation of the PI3K/Akt1 pathway and makes tumor cells highly sensitive to PARPi and PARPi/ionizing radiation (IR) combination. We showed that PI3K/Akt1 pathway plays an important role in the sensitization of cancer cell lines with compromised function of ARID1A to PARPi treatment.

**Discussion:**

We believe that using of PARPi monotherapy or in combination with radiation therapy is an appealing strategy for treating ARID1A-mutated cancers, as well as many other types of PI3K/Akt1-driven cancers.

## Introduction

The recent development of poly(ADP-ribose) polymerase inhibitors (PARPi) results from over 45 years of studies. Poly(ADP-ribose) polymerase (PARP) is an enzyme essential for the DNA single-strand breaks (SSB) repair (1–4). When the activity of PARP1 or PARP2 is compromised, DNA SSB lesions are unresolved and can be converted to DNA double-strand breaks (DSBs) by the cellular transcription mechanisms (5). Breast cancer susceptibility protein 1 (BRCA1) plays a significant role in the error-free homologous recombination repair (HRR) of DNA DSBs. Loss of BRCA1 activity leads to a switch from error-free HRR to error-prone non-homologous end-joining (NHEJ) repair of DSB DNA which resulted in genomic instability (6–8). The PARP/BRCA genetic interaction was described as the synthetic lethality effect: both genes when individually downregulated do not affect the cell survival, but a contemporary loss of both genes’ activities leads to cell death (9–11). Mechanisms of synthetic lethality can be applied to the treatment of cancer (12) and the PARP/BRCA genetic interaction demonstrated an effective approach for the treatment of ovarian cancer (OC) (13–16). However, inherited *BRCA1/2* mutations are present in 13-15% of OC (17), most frequently (18% in high-grade serous carcinomas) and less commonly for other histologic subtypes (18) and many cancer patients with *BRCA1/2-*wt tumors, sensitization to the different DNA-damaging agents with PARPi is less effective. Enhancing the efficacy of PARPi in the treatment of those OC without pathogenic mutations in homologous recombination deficiency proteins is an unmet clinical need.

*ARID1A* (also called BAF250a) is an important component of the mammalian Switch/Sucrose Non-Fermentable (SWI/SNF) chromatin-remodeling complex that regulates gene expression by controlling gene accessibility. *ARID1A* has been identified as a tumor suppressor gene with one of the highest mutation rates across different cancer types. Among OCs, *ARID1A* is mutated in >50% of clear-cell carcinomas (OCCC) and >30% of endometrioid carcinomas (19–21). Different reports demonstrated that ARID1A deficiency sensitizes cells to the PARPi (22, 23), however, the mechanism of this effect was not described. Previous investigations showed that ADIR1A protein plays a significant inhibitory role in the PI3K-Akt1 pathway (24–26). Furthermore, *ARID1A* mutations were discovered to occur frequently in a synergistic fashion with mutations in *PIK3CA* (26–28). Overactivation of the PI3K-Akt1 pathway suppresses the HRR of DNA DSBs by different mechanisms. As previously shown, Akt overactivation can suppress HRR *via* p70S6 kinase-dependent downregulation of MRE11 (29). It has also been demonstrated that stimulation of the PI3K-Akt1 pathway suppresses HRR *via* cytoplasmic retention of Rad51 and BRCA1 proteins (30, 31). These findings indicate that tumors with mutant *ARID1A* or ARID1A protein deficiency may depend more on the overactivated PI3K/AKT pathway than on homologous recombination for DNA repair. In the present work, we have demonstrated that downregulated or mutated ARID1A attenuates DNA HRR by stimulating the PI3K/Akt1 pathway and renders tumor cells highly sensitive to PARPi. We have also shown that the use of PARPi in combination with a DNA-damaging agent (for example - ionizing radiation (IR)) leads to a synthetic lethality effect, resulting in a significantly more pronounced therapeutic effect compared to monotherapy with PAPRi or a DNA-damaging agent. The results of our study provide a new strategy for using PARPi alone or in combination with DNA-damaging agents (radio or chemotherapy) to treat ARID1A-mutated cancers, as well as many other PI3K/Akt1-induced cancers.

## Materials and methods

### Cell culture, apoptosis, and clonogenic assays

OC cell lines CAOV-3, OVCA-429, SKOV-3, and TOV-21G were obtained from American Type Culture Collection (ATCC) and grown according to ATCC recommendations. The base medium for CAOV-3 – DMEM (cat.# 12491015, ThermoFisher), OVCA-429 – DMEM (with high glucose) (cat.# 11960044, ThermoFisher), and SKOV-3 was grown in McCoy’s 5a medium (cat.# 16600082, ThermoFisher). Medium for all three cell lines contained 10% fetal bovine serum (FBS) (cat.# A5256701, ThermoFisher), and 1% Penicillin/Streptomycin. The base medium for the TOV-21G cell line was a 1:1 mixture of MCDB 105 medium containing a final concentration of 1.5 g/L sodium bicarbonate and Medium 199 containing a final concentration of 2.2 g/L sodium bicarbonate with 15% of FBS and 1% Penicillin/Streptomycin (cat.# 15140122, ThermoFisher). For the clonogenic assay, cells were seeded into a 6-well plate or 60-mm culture dish. After an incubation period of 2 weeks, the colonies were fixed with methanol and stained with crystal violet. Cell apoptosis was determined through APC Annexin V/Propidium Iodide (PI) assay staining by using the APC-Annexin V Apoptosis Detection Kit (cat.# 640932, BioLegend) according to the manufacturer’s protocol. At specific time points, cells were stained and analyzed by flow cytometry (FACS Canto II flow cytometer, BD Biosciences).

### Antibodies, reagents, siRNAs

Primary antibodies used for Western blotting: anti-Akt-1 (1:1000 dilution, cat.# 4691, Cell Signaling); anti-phospho-Akt-1 (S473) (1:1000 dilution, cat.# 9018, Cell Signaling); anti-phospho-Akt-1 (T308) (1:1000 dilution, cat. 13038, Cell Signaling); anti-β-tubulin (1:1000 dilution, cat. 2128, Cell Signaling); anti-ARID1A (1:1000 dilution, cat.# NB100-55334, from Novus Biologicals). PARPi were purchased: ABT-888 from Enzo Life Sciences (cat.# ALX-270-444-M001); Olaparib from Selleck Chemical LLC (cat.# S1060). An inhibitor of PI3K LY294002 was obtained from Selleck Chemical LLC (cat.# S1105) and allosteric Akt inhibitor MK-2206 from Cayman Chemical (cat.# 11593). ARID1A Silencer™ siRNA and Silencer™ Select Negative siRNA Control were purchased from ThermoFisher Scientific (cat.# 4392420 and 4390843). Lipofectamine RNAiMAX (cat.# 13778075, ThermoFisher Scientific) was used for siRNA transfection according to manufacturer recommendations.

### Fluorescence-based DSB repair assay, adenovirus treatment, and flow cytometry

**Figure 1:**
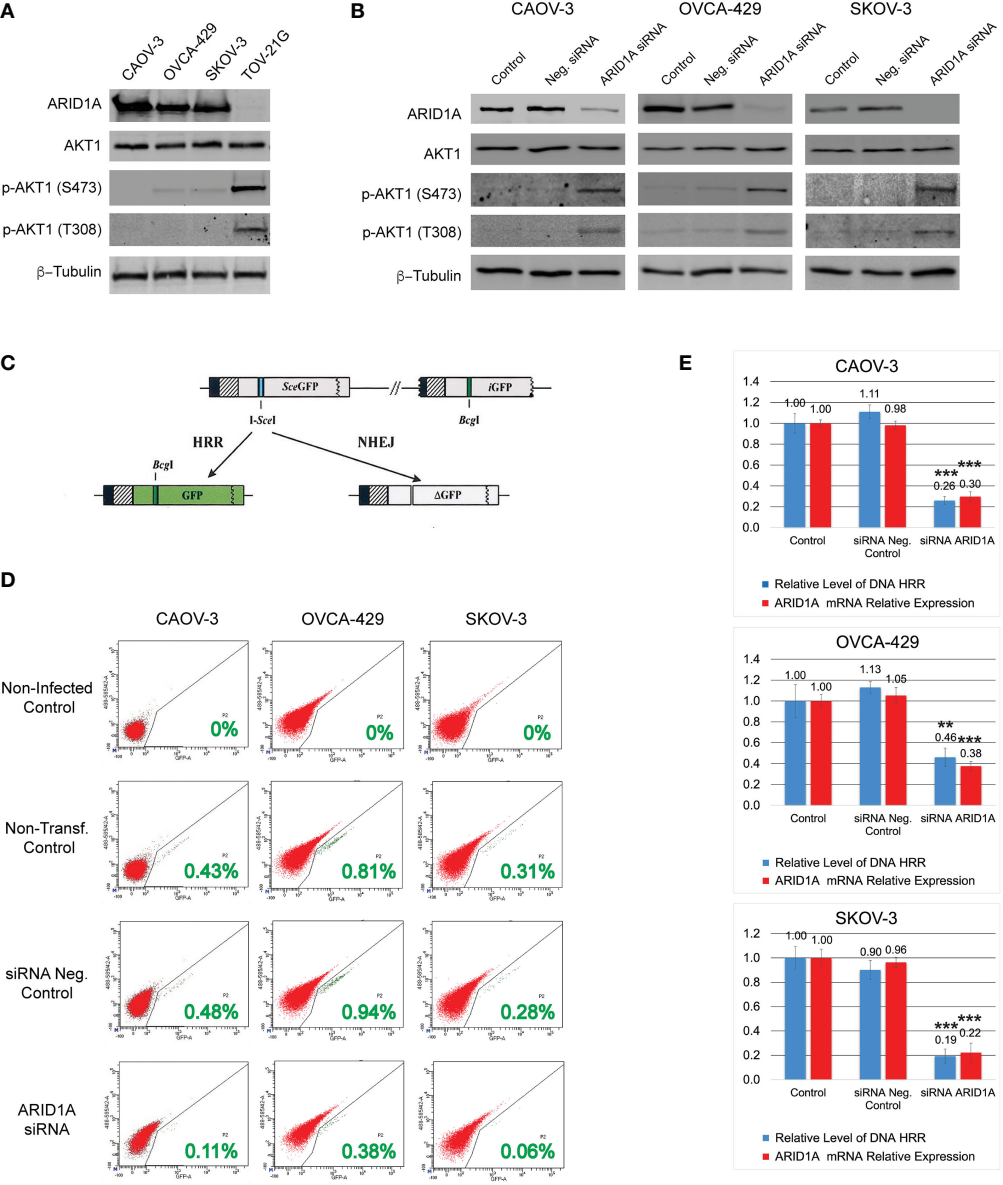
DNA HRR level depends on ARID1A protein expression. **(A)** Levels of ARID1A protein, total Akt-1, and phosphorylated forms of Akt-1 protein were assessed by Western blotting. Blotting with an anti-β-Tubulin antibody was used as a loading control. **(B)** Levels of ARID1A protein, total Akt1, and phosphorylated forms of Akt1 protein 24 h after transfection with 20nM of ARID1A siRNA or 20nM of Negative Control siRNA. **(C)** Schematic drawing of the Homologous Recombination Repair assay: The direct repeat GFP (DR-GFP) repair substrate contains a mutated *GFP* gene with an in-frame stop codon and an I-SceI site inserted (shown as a blue line) and a truncated *GFP* gene. Cleavage of the I-SceI site *in vivo* and repair by HR directed by the downstream *iGFP* repeat results in GFP^+^ cells. **(D)** CAOV-3, OVCA-429, and SKOV-3 cell lines were transfected with 20nM of ARID1A siRNA or 20nM of Negative Control siRNA. 24 hours after transfection all cells were infected with Ad-SceI-NG adenovirus. After 48 hours of infection, flow cytometry was used to determine the fraction of GFP^+^ cells in each sample. Non-transfected cells infected with Ad-SceI-NG were used as a non-transfected positive control. Cells transfected with 20nM of Negative Control siRNA were used as a transfected negative control. Non-transfected non-infected cells were used as a negative control. Green numbers represent the level of HRR. **(E)** Correlation between ARID1A mRNA expression and the level of HRR. Part of the CAOV-3, OVCA-429, and SKOV-3 cell lines treated as described in **(C)** were subjected to qPCR analysis of endogenous *ARID1A* mRNA expression 24 h after transfection with 20nM of ARID1A siRNA or 20nM of Negative Control siRNA. Results are presented as the mean ± SD of 3 independent experiments. The *P-values* were calculated with the Student t-test and shown as ** – p < 0.05, *** – p < 0.001.

All cell lines used in our study were stably transfected with the pDR-GFP reporter construct. The reporter constructs and the fluorescence-based assay for measuring the frequency of HRR at a single chromosomal DSB have been described previously (32, 33). Infection with an I-SceI expression adenovirus (Ad-SceI-NG) produces a DNA DSB in the SceGFP sequence that can be repaired by 2 general mechanisms: error-free HRR or error-prone non-homologous end joining repair (NHEJ). In this assay, a functional GFP sequence can only be restored if the DSB is repaired by HRR in an error-free manner using the downstream GFP fragment (iGFP) as a template (**Figure 1C**). The percentage of GFP-positive cells after infection with Ad-SceI-NG represents the level of HRR in the test. The Ad-SceI-NG adenovirus was a generous gift from Dr. Kristoffer Valerie (32, 33). Adenovirus was added to the culture medium at 30 virus particles/cell and incubated while rocking for 4 h at 37°C. The virus was then removed, and a fresh medium was applied. After 48 hours of infection, flow cytometry was used to determine the fraction of GFP-positive cells in each sample. The amount of HRR was calculated as a percentage of the GFP-positive cells in 30,000 cells counted.

### Western blotting

Proteins were separated by SDS-PAGE and transferred to nitrocellulose membranes. The membranes were exposed to antibodies at specific dilutions. Specific protein bands were detected using infrared-emitting conjugated secondary antibodies: anti-Rabbit DyLight™800 4XPEG Conjugate (1:10,000 dilution, cat.# 5151, Cell Signaling), and the ChemiDoc™ MP Imaging System (Bio-Rad).

### RNA extraction, real-time quantitative PCR

Total RNA was isolated from the cultured cells following the manufacturer’s instructions with the RNeasy kit (cat.# 74004, QIAGEN). The RNA concentration was measured using a NanoDrop ND-1000 spectrophotometer (Thermo Scientific, Wilmington DE). RNA purity was assessed by the ratios of A260/A280 and A260/A230. RNA integrity was evaluated by the ratio of 28S/18S ribosomal RNA (rRNA) and the RNA integrity number (RIN) using an Agilent 2100 BioAnalyzer (Agilent Technologies, Wilmington DE). cDNA synthesis and genomic DNA elimination were performed by using RT^2^ First Strand Kit (cat.# 330404, QIAGEN). Samples were amplified using RT^2^ SYBR^®^ Green qPCR Mastermix probes with ROX (carboxy-X-rhodamine) passive reference dye from QIAGEN (cat.# 330529)on the QuantStudio 3 RT-PCR machine (Applied Biosystems). The real-time PCR data were normalized by ROX passive reference. The specificity of amplicons was verified by melting curve analysis and C_t_ values of all amplicons were normalized using mRNA expression as an internal control. The 2^-ΔΔCt^ method was used for the calculation of relative mRNA expression levels. The following RT^2^ qPCR Primer Assays (QIAGEN) were used: RT² qPCR Primer Assay for Human ACTB (GeneGlobe ID: PPH00073G-200), RT² qPCR Primer Assay for Human ARID1A (GeneGlobe ID: PPH13453B-200).

### Statistical analysis

Unless indicated otherwise, data are demonstrated as mean ± standard deviation (SD). The significance of the difference between groups was determined by paired or unpaired two-tailed Student’s t-test or the one-way analysis of variance (ANOVA). The Data Analysis Toolpak for Excel was used for the calculation of the Student’s t-test or the ANOVA. Differences were considered significant for *p*-values < 0.05.

## Results

### Mutation or downregulation of ARID1A expression stimulates PI3K/Akt1 pathway and reduces HRR of DNA DSB

Four different OC cell lines were used in our study. Three cell lines contain a wild type of *ARID1A* gene: CAOV-3, OVCA-429, and SCOV-3; whereas the TOV-21G cell line contains a mutant *ARID1A* gene leading to loss of the protein expression (**Figure 1A**). Since the ARID1A protein is involved in the regulation of the PI3K/Akt1 pathway, we tested the protein expression level and phosphorylation status of Akt1. All four cell lines demonstrated similar levels of total Akt1, however, TOV-21G showed a significant increase in Akt-1 phosphorylation at S473 and T308 (**Figure 1A**). In TOV-21G cells the *ARID1A* mutation coexists with a *PIK3CA* mutation which can additionally stimulate PI3K/Akt1 pathway. Hence, as a next step, we tested if ARID1A protein expression alone can modulate the activity of the PI3K/Akt1 pathway. All three cell lines with wild-type *ARID1A* were transfected with 20nM of siRNA against ARID1A or 20nM of negative control siRNA. As shown in **Figure 1B**, downregulation of ARID1A protein expression stimulated phosphorylation of Akt-1 protein on S473 and T308. Both experiments demonstrated a strong connection between the level of ARID1A protein expression and PI3K/Akt1 pathway activation evaluated by Akt-1 protein phosphorylation.

It was previously demonstrated that stimulation of the PI3K/Akt1 pathway suppresses the HRR of DNA DSB by various mechanisms (29–31). To estimate the impact of ARID1A on the level of DNA HRR, all cell lines were stably transfected with the DR-GFP reporter construct. Infection of stably transfected cell lines with an I-SceI expression adenovirus (Ad-SceI-NG) generates a DSB in the SceGFP sequence that can be repaired by 2 general mechanisms: HRR or NHEJ. In this assay, a functional GFP sequence can only be restored if the DSB is repaired in an error-free manner using the downstream GFP fragment (iGFP) as a template for HRR (**Figure 1C**). The percentage of GFP-positive cells after infection with Ad-SceI-NG represents the level of DNA HRR in the test (**Figure 1D**). Transfection with the negative control siRNA did not significantly affect the level of DNA HRR for all three cell lines with wild type of *ARID1A* (**Figures 1D, E**). However, the reduction of *ARID1A* expression by siRNA transfection significantly reduces the relative level of DNA HRR: CAOV-3 – 0.26±0.039 (ARID1A siRNA transfection) *vs.* 1.11±0.065 (Neg. control siRNA transfection), *p<0.001*; OVCA-429 – 0.46±0.089 (ARID1A siRNA transfection) *vs.* 1.13±0.055 (Neg. control siRNA transfection), *p<0.03*; SKOV-3 – 0.194±0.057 (ARID1A siRNA transfection) *vs.* 0.9±0.093 (Neg. control siRNA transfection), *p<0.001* (**Figure 1E**).

**Figure 2:**
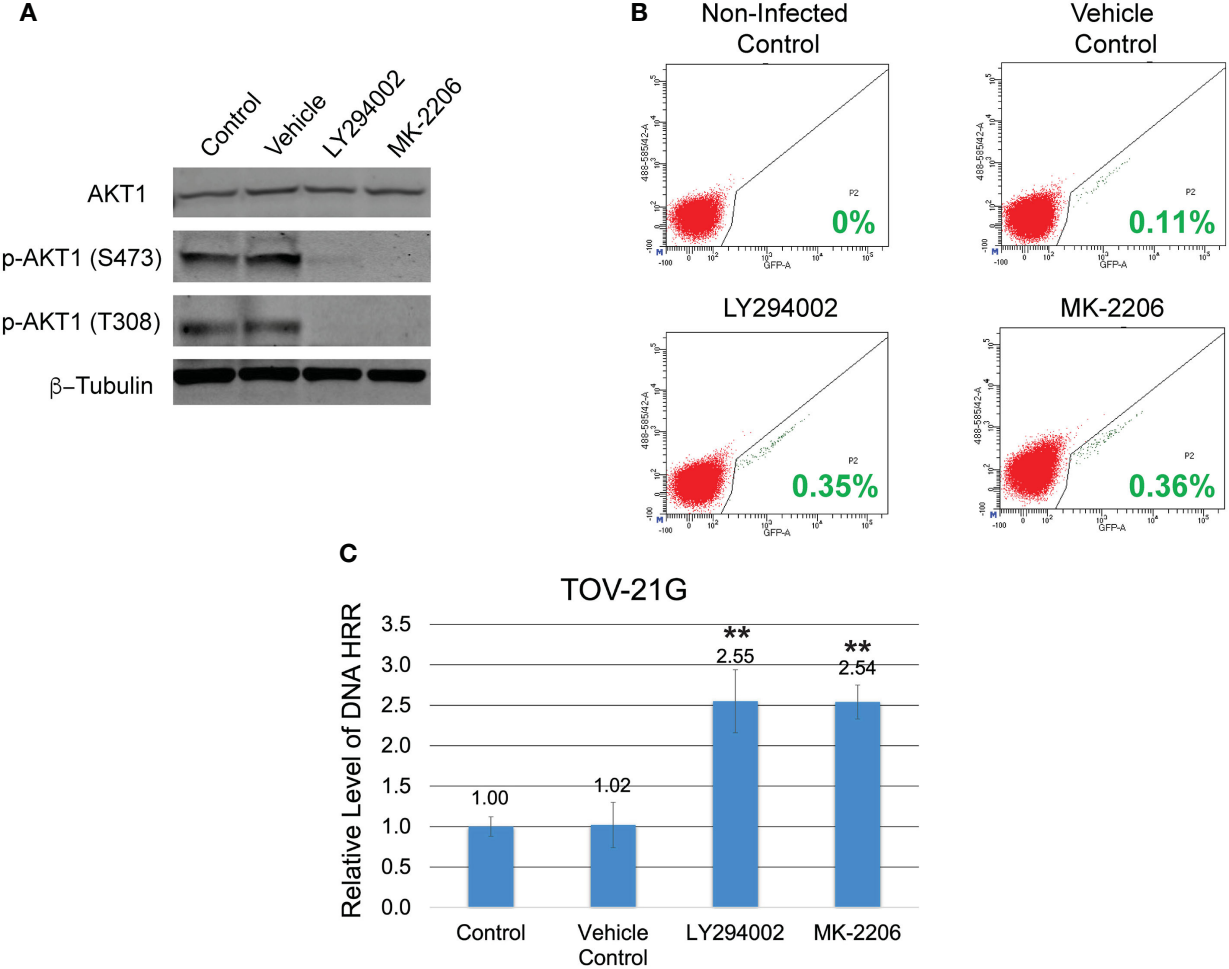
Inhibition of the PI3K/Akt1 pathway reinforces the level of DNA HRR in the TOV-21G cell line. **(A)** TOV-21G cell line was treated with a potent inhibitor of PI3K LY294002 (in a dose of 50μM), selective Akt inhibitor MK-2206 (in a dose of 2μM), or a vehicle (DMSO). After 6 h, levels of total Akt-1 and phosphorylated forms of Akt-1 protein were assessed with Western blotting. **(B)** After 6 h of incubation with LY294002 or MK-2206 media was changed to the fresh one and TOV-21G cells were infected with Ad-SceI-NG adenovirus. After 48 hours of infection, flow cytometry was used to determine the fraction of GFP^+^ cells in each sample. Cells incubated with the same amount of a vehicle (DMSO) were used as a vehicle positive control. Non-treated non-infected cells were used as a negative control. Green numbers represent the level of HRR. **(C)** HRR level of the TOV-21G cell line was normalized to the non-treated positive control and shown as a relative expression level. Results are presented as the mean ± SD of 3 independent experiments. The *P-values* were calculated with the Student t-test and shown as ** – p < 0.0001.

The coexistence of *ARID1A* and *PIK3CA* mutations in TOV-21G cells leads to PI3K/Akt1 pathway overactivation (**Figure 1A**) and may result in a substantial decline in DNA HRR activity. To test the correlation between the level of DNA HRR and PI3K/Akt1 pathway activity, TOV-21G cells stably transfected with DR-GFP reporter construct were treated with a potent inhibitor of PI3K LY294002 or selective Akt inhibitor MK-2206. Both inhibitors dramatically blocked Akt1 phosphorylation on S473 and T308 (**Figure 2A**) and significantly enhanced DNA HRR activity (**Figures 2B, C**) Relative DNA HRR for TOV-21G cell line: Control = 1.0±0.097; Vehicle control = 1.02±0.284 (*p=0.797)*; LY294002 treatment = 2.55±0.365 (*p<0.0001)*; MK-2206 treatment = 2.54±0.217 (*p<0.0001)*. Thus ARID1A/PI3K/Akt1 vector significantly affects the ability of cells to repair the DNA DSBs through the mechanisms of HRR.

### ARID1A status and sensitivity to PARPi

**Figure 3:**
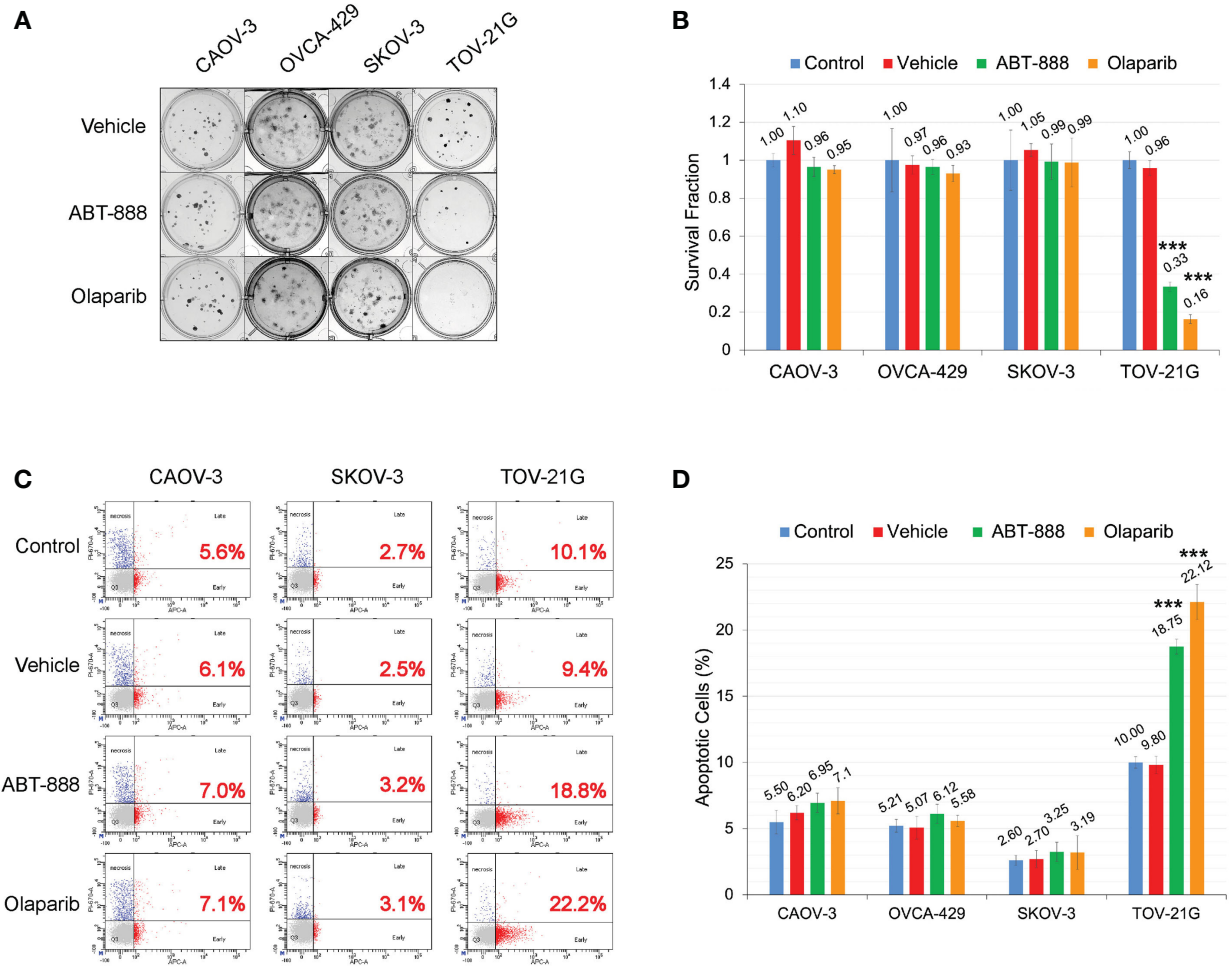
ARID1A status and sensitivity to PARP-inhibitors ABT-888 and Olaparib. **(A)** Clonogenic analysis of cell lines treated with 10μM ABT-888 or 1μM Olaparib for 24 h and a vehicle (DMSO). **(B)** Results of the clonogenic analysis were normalized to non-treated positive control and presented as the mean ± SD for quadruplicate samples. The *P*-value was calculated with the ANOVA t-test and shown as *** – p < 0.001. **(C)** Cells treated as described in **(A)** and apoptosis assessed by AnnexinV FITC/Propidium iodide staining and flow cytometric analysis 48 h after the start of incubation. Red numbers represent the percentage of apoptotic cells (early + late apoptosis). **(D)** Summary of flow cytometry analysis of apoptosis. Columns represent the means ± SD values for apoptotic cells obtained from three individual experiments. For **(B, D)**, the *P-values* were calculated with the ANOVA test and shown as *** – p < 0.001.

All cell lines were subjected to the clonogenic assay with two different PARPi ABT-888 and Olaparib. Survival fraction was estimated for 4 different groups: 1) Non-treated control; 2) Vehicle control (with DMSO); 3) 10μM of ABT-888; 4) 1μM of Olaparib. All cell lines were incubated with the PARPi or vehicle for 24h, and then culture media were replaced with fresh media without drug or vehicle. Only TOV-21G cells demonstrated a significant decrease in clonogenic survival with ABT-888 and Olaparib treatment (**Figures 3A, B**): ABT-888 treatment – 0.33±0.025, *p<0.001;* Olaparib treatment – 0.16±0.023, *p<0.001.* Similar results were obtained when apoptosis was assessed after treatment with ABT-888 and Olaparib (**Figure 3C**). Levels of apoptosis were measured by the APC AnnexinV/PI assay after 48 h of incubation with PARPi. As in the clonogenic experiment, only TOV-21G cells show a significant increase in apoptosis after treatment with PARPi (**Figure 3D**): ABT-888 – 18.75±0.56% (*p<0.001*) and Olaparib – 22.12±1.33% (*p<0.001*) *vs.* Vehicle control – 9.8±0.66%.

### ARID1A status and sensitization to ionizing radiation by PARPi

**Figure 4:**
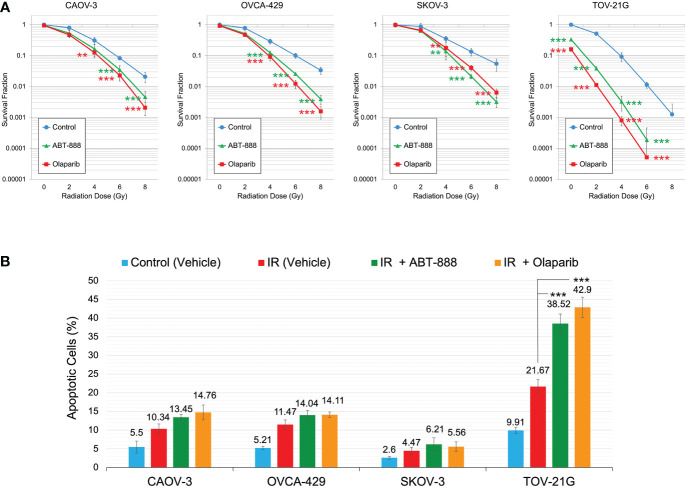
Correlation of ARID1A expression status with sensitization to IR by PARPi treatment. **(A)** Cell lines were pre-treated with 10μM ABT-888, 1μM Olaparib, or a vehicle (DMSO) and were subjected to the clonogenic assay with different doses of IR. Results of the clonogenic analysis were normalized to non-irradiated vehicle control and presented as the mean ± SD for quadruplicate samples. **(B)** Cell lines were treated as described in Figure 4A and apoptosis was assessed 48 hr after 2 Gy IR as described in Figure 3. Columns represent the means ± SD values for apoptotic cells obtained from three individual experiments. For **(A, B)**, the *P-values* were calculated with the ANOVA test and shown as ** – p < 0.05 and *** – p < 0.001.

**Figure 5:**
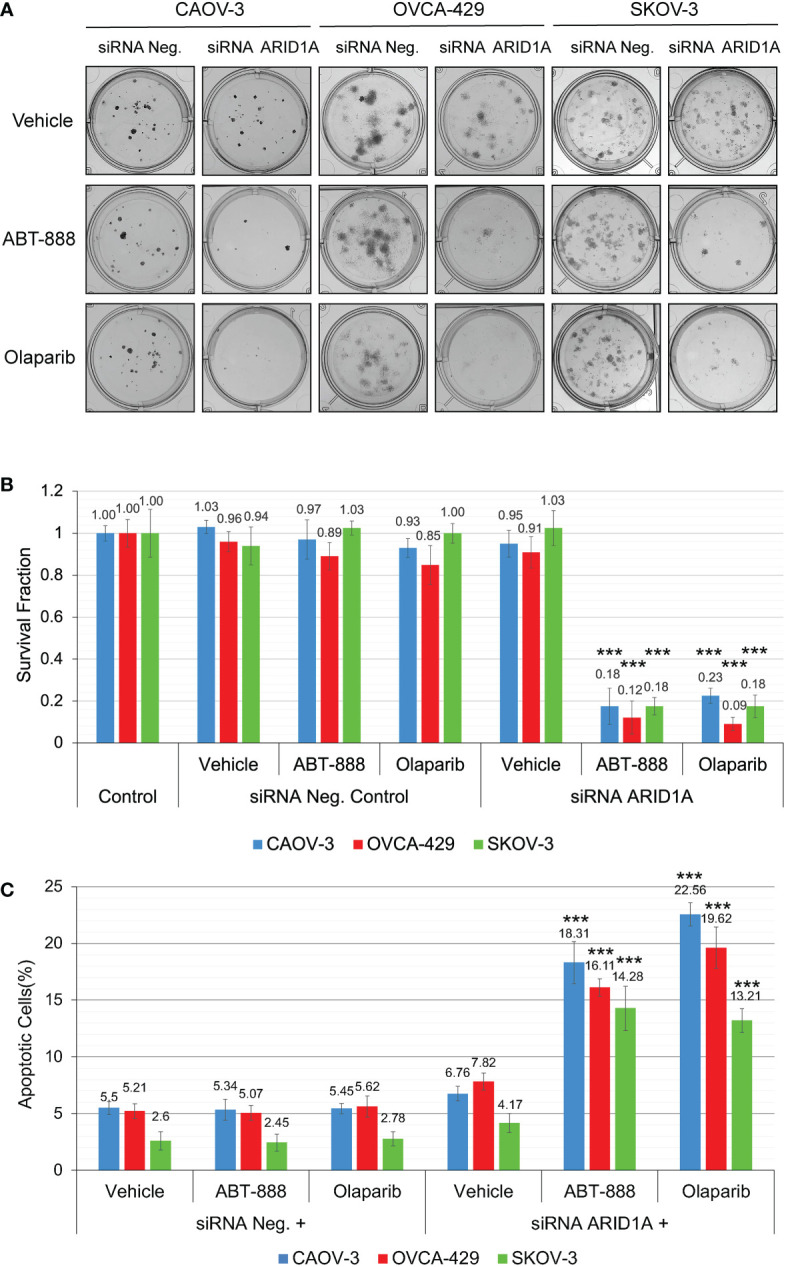
Reduction of *ARID1A* expression stimulates sensitivity to PARPi ABT-888 and Olaparib. **(A)** Clonogenic analysis of CAOV-3, OVCA-429, and SKOV-3 cells transfected with 20 nM of *ARID1A* siRNA and treated with 10μM ABT-888, 1μM Olaparib, or the same amount of vehicle (DMSO). Cells transfected with 20nM of Negative Control siRNA were used as a negative control. **(B)** Results of the clonogenic analysis were normalized to non-treated control and presented as the mean ± SD for quadruplicate samples. **(C)** Cell lines were treated as described in **(A)** and apoptosis was measured at 48 h after the start of incubation with ABT-888, Olaparib, or Vehicle (DMSO) by methods described in **Figure 3**. Columns represent the means ± SD values for apoptotic cells obtained from three individual experiments. For **(B, C)**, the *P-value*s were calculated with the ANOVA test and shown as *** – p < 0.001.

All cell lines were subjected to the clonogenic assay with radiation doses: 0, 2, 4, 6, and 8 Gy. ABT-888 or Olaparib were applied 4 h before IR. The cell media was replaced 24 h post-IR with the fresh media without drugs or vehicle added. Survival fraction was estimated for 3 treatment groups: 1) Vehicle control (with DMSO); 2) 10 μM ABT-888; 3) 1 μM Olaparib (**Figure 4A**). For cell lines with wild type of *ARID1A* (CAOV-3, OVCA-429, and SKOV-3), treatment with ABT-888 or Olaparib demonstrated significant increases of sensitization to doses of IR >4 Gy. For wild-type cell lines, PARPi didn’t stimulate sensitization to 2 Gy, and only the OVCA-429 cell line demonstrated statistically significant sensitization to 4 Gy by ABT-888 or Olaparib treatment (Control = 0.28±0.054 *vs.* ABT-888 = 0.125±0.018 *vs.* Olaparib = 0.09±0.025). In contrast, ABT-888 and Olaparib were potent radiosensitizers of TOV-21G even at 2 Gy (Control = 0.51±0.051 *vs.* ABT-888 = 0.039±0.005 *vs.* Olaparib = 0.011±0.001) (**Figure 4A**). Similar results were obtained in apoptosis assays (**Figure 4B**). Pre-treatment with ABT-888 or Olaparib did not significantly enhance apoptosis after 2 Gy radiation exposure in any of the cell lines except for the TOV-21G. Apoptosis level for TOV-21G cell line was: IR+Vehicle = 21.67±1.96%; IR+ABT-888 = 38.52±2.57 (*p<0.001*); IR+Olaparib = 42.9±2.73 (*p<0.001*). *Effect of ARID1A expression level on the sensitivity of wild-type ARID1A cell lines to PARPi*: A decline of the cell’s DNA HRR efficiency increases sensitivity to PARPi by the synthetic lethality mechanism. Experiments in **Figures 1D, E** demonstrate significant attenuation of DNA HRR for CAOV-3, OVCA-429, and SKOV-3 cell lines in response to inhibition of *ARID1A* expression level by siRNA approach. We next tested how the downregulation of *ARID1A* expression affects the sensitivity of wild-type *ARID1A* cell lines to PARPi. CAOV-3, OVCA-429, and SKOV-3 cells were transfected with 20nM of ARID1A siRNA or 20nM of negative control siRNA. ABT-888 or Olaparib was applied 24 h after transfection and 24 hrs later culture media were replaced with fresh media not containing the drug or vehicle. The clonogenic assay demonstrated significant decreases in survival for cells transfected with ARID1A siRNA and treated with ABT-888 or Olaparib: CAOV-3 – 0.18±0.009 for ABT-888 and 0.23±0.036 for Olaparib *(both p<0.001);* OVCA-429 – 0.12±0.079 for ABT-888 and 0.09±0.032 for Olaparib *(both p<0.001);* SKOV-3 – 0.18±0.041 for ABT-888 and 0.18±0.054 for Olaparib *(both p<0.001)* (**Figures 5A, B**). Similar results were obtained when cell killing was measured by apoptosis (**Figure 5C**).

### Inhibition of PI3K/Akt1 pathway by LY294002 or MK-2206 attenuates sensitivity of TOV-21G cells to PARPi

**Figure 6:**
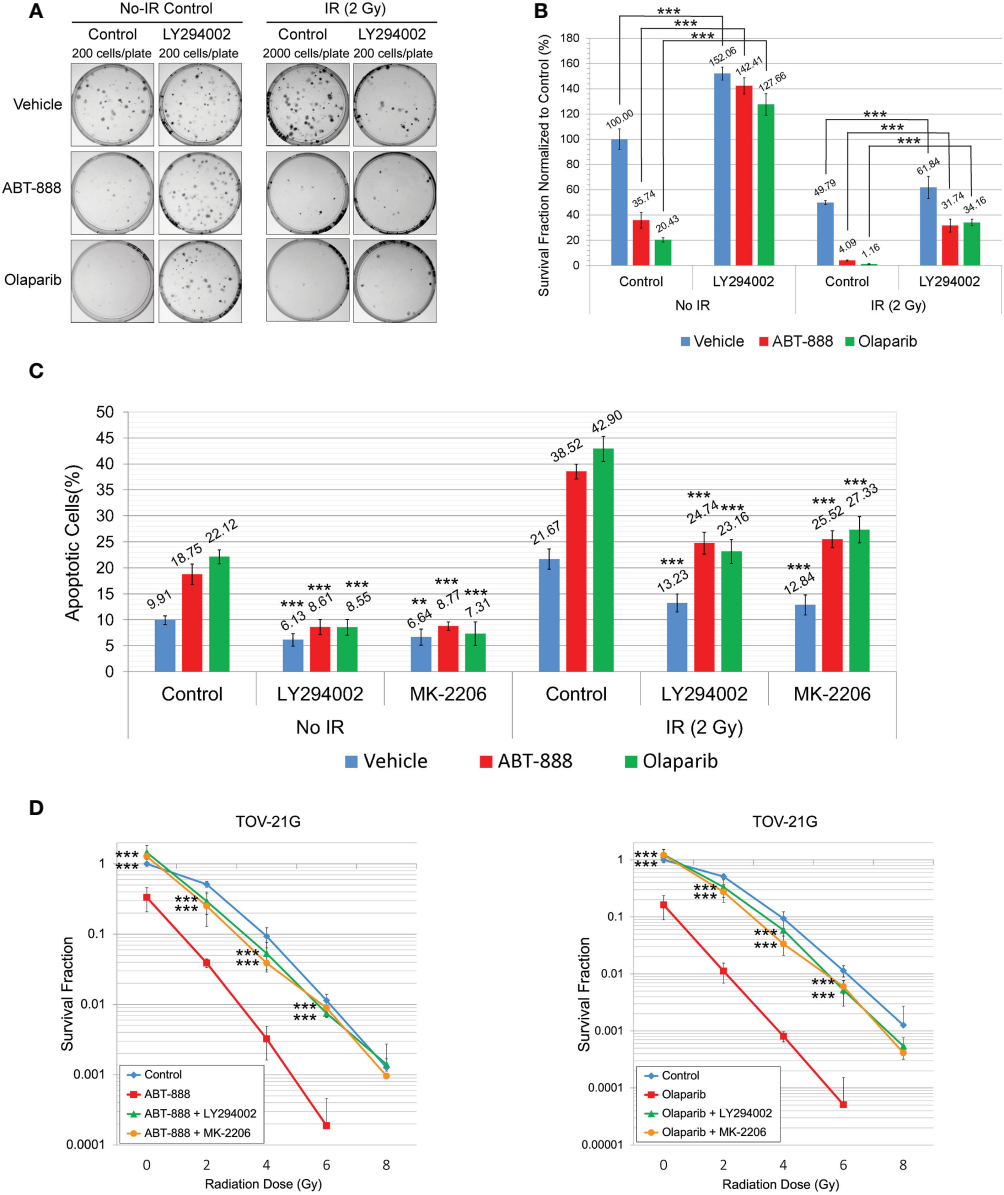
Pretreatment with LY294002 or MK-2206 attenuates the sensitivity of the TOV-21G cell line to PARPi and PARPi/IR combination. **(A)** Clonogenic analysis of TOV-21G cells pre-treated with 50μM LY294002 and subjected to treatment with 10μM ABT-888, 1μM Olaparib, their combination, or vehicle (DMSO) with 2 Gy of IR. **(B)** Results of the clonogenic analysis were normalized to non-treated control and presented as the mean ± SD for quadruplicate samples. **(C)** TOV-21 cell line was pre-treated with 50μM LY294002, 2 μM MK-2206, or vehicle (DMSO). After 6 h of pre-treatment media was changed to the fresh one and cells were incubated with the vehicle (DMSO), 10 μM ABT-888, or 1 μM Olaparib for the next 6 h. 48h after the start of incubation cells were assayed for apoptosis as described in Figure 3. Columns represent the means ± SD values for apoptotic cells obtained from three individual experiments. **(D)** Clonogenic analysis of TOV-21G cells pre-treated with 50μM LY294002 or 2μM MK-2206 and subjected to treatment with 10μM ABT-888, 1μM Olaparib, or their combination with different doses of IR. Results of the clonogenic analysis were normalized to non-irradiated vehicle control and presented as the mean ± SD for quadruplicate samples. The *P-values* were calculated for **(B)** with the Student t-test, for **(C, D)**, with the ANOVA test and shown as ** – p < 0.05, *** – p < 0.001.

In experiments in **Figure 2**, we demonstrated that incubation of TOV-21G cells with a potent inhibitor of PI3K LY294002 or selective Akt inhibitor MK-2206 blocked Akt-1 phosphorylation on S473 and T308 (**Figure 2A**) and significantly increased DNA HRR level. We next tested how inhibition of the PI3K/Akt1 pathway affects the sensitivity of the TOV-21G cell line to PARPi and PARPi/IR combination. TOV-21 cells were pre-treated with 50μM LY294002 or the same volume as a vehicle (DMSO). After 6 hr media was replaced with the fresh media containing either vehicle (DMSO), 10 μM ABT-888, or 1 μM Olaparib, and cells were incubated for another 6 h, and then cells were irradiated at 2 Gy. Survival fraction was estimated by clonogenic assay (**Figures 6A, B**). Pretreatment with LY294002 significantly increased the survival fraction for nonirradiated cells treated with ABT-888 (Vehicle – 35.74±6.14 *vs.* LY294002 – 142.41±6.44) or Olaparib (Vehicle – 20.43±1.70 *vs.* LY294002 – 127.66±8.51) as well as for cells received 2 Gy of IR in combination with ABT-888 (Vehicle – 4.09±0.45 *vs.* LY294002 – 31.74±5.11) or Olaparib (Vehicle – 1.16±0.43 *vs.* LY294002 – 34.16±2.60). Of potential interest is the finding that control cells treated with the vehicle also sowed enhanced survival LY294002 pretreatment (No-radiation group: Vehicle – 100±8.15 *vs.* LY294002 – 152.06±5.20; Irradiated group (2 Gy): Vehicle – 49.79±1.56 *vs.* LY294002 – 61.84±8.73) (**Figure 6B**).

Apoptosis was also assessed at 48 h after the start of pretreatment with LY294002 (50μM) or MK-2206 (2 μM) (**Figure 6C**). Both drugs significantly attenuated effects of ABT-888 and Olaparib on apoptosis in non-irradiated cells (Vehicle+ABT-888 – 18.75±1.97% *vs.* LY294002+ABT-888 – 8.61±1.44% *vs.* MK-2206+ABT-888 – 8.77±0.83%; Vehicle+Olaparib – 22.12±1.33% *vs.* LY294002+Olaparib – 8.55±1.51% *vs.* MK-2206+Olaparib – 7.31±2.29%) and in cells received 2 Gy of IR (Vehicle+ABT-888 – 38.52±1.45% *vs.* LY294002+ABT-888 – 24.74±2.11% *vs.* MK-2206+ABT-888 – 25.52±1.63%; Vehicle+Olaparib – 42.9±2.43% *vs.* LY294002+Olaparib – 23.16±2.29% *vs.* MK-2206+Olaparib – 27.33±2.53%). Pretreatment with LY294002 or MK-2206 was also able to significantly decrease apoptosis in vehicle-treated cells (non-irradiated cells: Vehicle – 9.91±0.84% *vs.* LY294002 – 6.13±1.20% *vs.* MK-2206 – 6.64±1.53%; 2 Gy irradiated cells: Vehicle – 21.67±1.96% *vs.* LY294002 – 13.23±1.74% *vs.* MK-2206 – 12.84±1.93%) (**Figure 6C**). Clonogenic assay with different doses of IR demonstrated that pretreatment with LY294002 or MK-2206 significantly reduced the ability of ABT-888 and Olaparib to sensitize the TOV-21G cell line to IR (**Figure 6D**).

## Discussion

PARPi are the first drugs designed to use a synthetic lethality approach. The highest efficacy for PARPi has been demonstrated for OC patients with *BRCA* mutations. PARPi activity was also demonstrated for *BRCA*wt cancers due to mutations in genes critical for DNA repair (e.g., ATM, BARD1, BRIP1, CHEK2, NBN, PALB2, RAD51C, and RAD51D) (34, 35). The idea that DNA HRR can be compromised by many different factors generated great interest in enhancing individualized profiling of homologous recombination deficiency (HRD) of OC to predict sensitivity to therapy with PARPi (34, 36, 37). Recently, DNA-based homologous recombination deficiency (HRD) score was developed on the basis of loss of heterozygosity (LOH), telomeric allelic imbalance (TAI), and large-scale state transitions (LST) (37)f. HRD is the first phenotypically defined predictive marker for therapy with PARPi in OC. As HRD assays are increasingly used for treatment planning, our goal is to characterize the molecular mechanisms behind the onset of HRD in patients with ovarian cancer and to identify novel predictive markers for treatment with PARPi-based therapy. Our previous work showed that cancer cells with HRD compensated this deficiency by activation of error-prone NHEJ (33).

A close correlation between *ARID1A* mutations and the activity of the PI3K/Akt1 signaling pathway has been previously described (38). In endometrial cancer (39), ovarian clear cell carcinoma (28), colon cancer (40), and gastric cancer (24), loss of ARID1A expression stimulates Akt1 phosphorylation. ARID1A protein activates SWI/SNF complex which inhibits PIK3CA and PDK1 transcription (24). Also, *ARID1A* mutations frequently occur with mutations in *PIK3CA* (26–28, 39, 41), leading to stimulation of the PI3K/Akt1 pathway synergistically. Hence, cancer cells with ARID1A mutation or deficiency depend more on the PI3K/Akt1 pathway than the cells expressing normal ARID1A. Previous investigations demonstrated that stimulation of the PI3K/Akt1 pathway suppresses the HRR of DNA DSBs by multiple mechanisms (29–31).

Our data show a high correlation between ARID1A protein expression level, PI3K/Akt1 pathway activity, and DNA HRR efficacy. Inhibition of ARID1A expression or its functional impairment due to mutation leads to stimulation of the PI3K/Akt1 pathway as shown by the increase in phosphorylation on S473 and T308 of Akt1 and subsequent impairment of DNA HRR mechanisms. In contrast, inhibiting the PI3K/Akt1 pathway by the potent inhibitor of PI3K LY294002 or selective Akt inhibitor MK-2206 significantly led to a recovery in DNA HRR in the *ARID1A* mutant TOV-21G cells. Treatment of wild-type *ARID1A* OC cell lines with PARPi ABT-888 or Olaparib did not affect survival fraction or apoptosis level. Only TOV-21G demonstrated significant stimulation of apoptosis and a decrease in survival fraction in response to PARPi in monotherapy. The same correlation was demonstrated when ABT-888 and Olaparib were used for sensitization to IR. Pretreatment of the TOV-21G cell line with PARPi demonstrated very high stimulation of sensitivity to all doses of IR. In contrast with the ARID1A wild-type CAOV-3, OVCA-429, and SKOV-3 cells PARPi were not effective radiosensitizers at 2 Gy of IR and only higher IR doses revealed sensitization by PARPi.

The importance of the ARID1A/PI3K/Akt1 pathway to PARPi sensitivity was demonstrated in our study by two approaches. First, we showed that the downregulation of ARID1A protein expression in CAOV-3, OVCA-429, and SKOV-3 cell lines significantly stimulated the sensitivity of these cells to PARPi ABT-888 and Olaparib. Second, inhibition of PI3K/Akt1 activity in the TOV-21G cell line by pre-treatment with LY294002 and MK-2206 revoked the sensitivity of this cell line to PARPi as well as abolished the sensitization effect of PARPi to IR. In summary, we demonstrated that *ARID1A* mutation stimulates PI3K/Akt1 pathway, attenuates DNA HRR, and makes tumor cells highly sensitive to PARPi and PARPi/IR combination.

Our study contains several limitations. First, only IR was used as a DNA-damaging agent. Further research into chemotherapeutic drugs as DNA-damaging agents is needed. Second, in our study, we use only the siRNA approach to decrease the expression of ARID1A in several cancer cell lines. In future studies, cell lines with knockout ARID1A and members of the PI3K/AKT pathway provide us with more information. Third, studying cell lines *in vitro* gives us only proof of principle. Animal model studies are needed to obtain more data.

The effect of *ARID1A* mutation can be compared with another commonly mutated gene in human cancers, *phosphatase and tensin homolog* (*PTEN*) (42). Both ARID1A and PTEN proteins share a common function of inhibition of the PI3K/Akt1 pathway. As for ARID1A, cells with mutated or depleted PTEN show over-activation of the PI3K/Akt1 pathway and impairment of DNA HRR (43–46). Consistently, PI3K/Akt1 inhibition restored DNA HRR efficiency in PTEN-depleted cells (47). As for ARID1A, the DNA HRR inefficiency caused by PTEN depletion or mutation, sensitizes tumor cells to PARPi, both *in vitro* and *in vivo* (43, 47).

Activation of the PI3K/Akt1 pathway can occur not only by mutations in PI3K, inactivating mutations or the loss of PTEN and ARID1A, but also mutations in other upstream oncogenes (e.g. RAS) (38, 48). According to current research, over 25% of lung adenocarcinoma mutations are RAS mutations (49), and the Kristin isoform of Ras (K-Ras) is mutated in over 90% of pancreatic ductal adenocarcinomas (50). As mutations in RAS are common to a variety of cancers many different strategies that aim to down-regulate constitutively active PI3K/Akt1 pathway have been explored (51, 52). We propose that another appealing strategy for treating cancers with RAS mutations, as well as many other types of PI3K/Akt1-driven cancers, is the use of PARPi in monotherapy or in combination with chemo- or radiation therapy.

*ARID1A* mutations, although uncommon in high-grade serous carcinoma of the ovary, are relatively common in cancers with clear cell and endometrioid histology (21, 53). As these subtypes of OC are relatively chemotherapy resistant (54), finding effective therapies is an unmet need. The use of PARPi in combination with different DNA-damaging agents can significantly reduce the resistance of these cancer subtypes to standard chemotherapy. In some cases, PARPi can also be used in combination with RT. The safety of treatment with PARPi in combination with RT in OC has been already demonstrated in several early-phase clinical trials (55, 56). Targeting this therapy to ARID1A mutant/deficient malignancies should be strongly considered in future clinical trials of enhancing PARPi efficacy for women without germline mutations in HR deficiency pathways and those with BRCAwt tumors with few therapeutic options.

## Conclusions

The present study demonstrates that mutated or downregulated ARID1A compromises the HRR of DNA DSBs through stimulation of the PI3K-Akt1 pathway. Attenuation of DNA HRR in ARID1A-mut tumor cells sensitizes them to PARPi and PARPi/IR combination by a mechanism of synthetic lethality.

## Data availability statement

The raw data supporting the conclusions of this article will be made available by the authors, without undue reservation.

## Author contributions

VY and ST participated in the conception and design of the study. Conceptualization: VY, EF, and ST. Methodology: VY. Validation: SS, EF, and ST. Formal analysis: VY, SS, EF, and ST. Writing-original draft preparation: VY. Writing- review and editing: SS, EF, and ST. Visualization and supervision: VY and ST. All authors contributed to the article and approved the submitted version.
