# Supplementary material for: PARP inhibitors in the treatment of ARID1A mutant ovarian clear cell cancer: PI3K/Akt1-dependent mechanism of synthetic lethality
Source: Front Oncol. 2023 Feb 22;13:1124147. doi: 10.3389/fonc.2023.1124147 (PMC9992988; doi:10.3389/fonc.2023.1124147)
Supplement: Supplementary file 1 [file DataSheet_1.pdf]

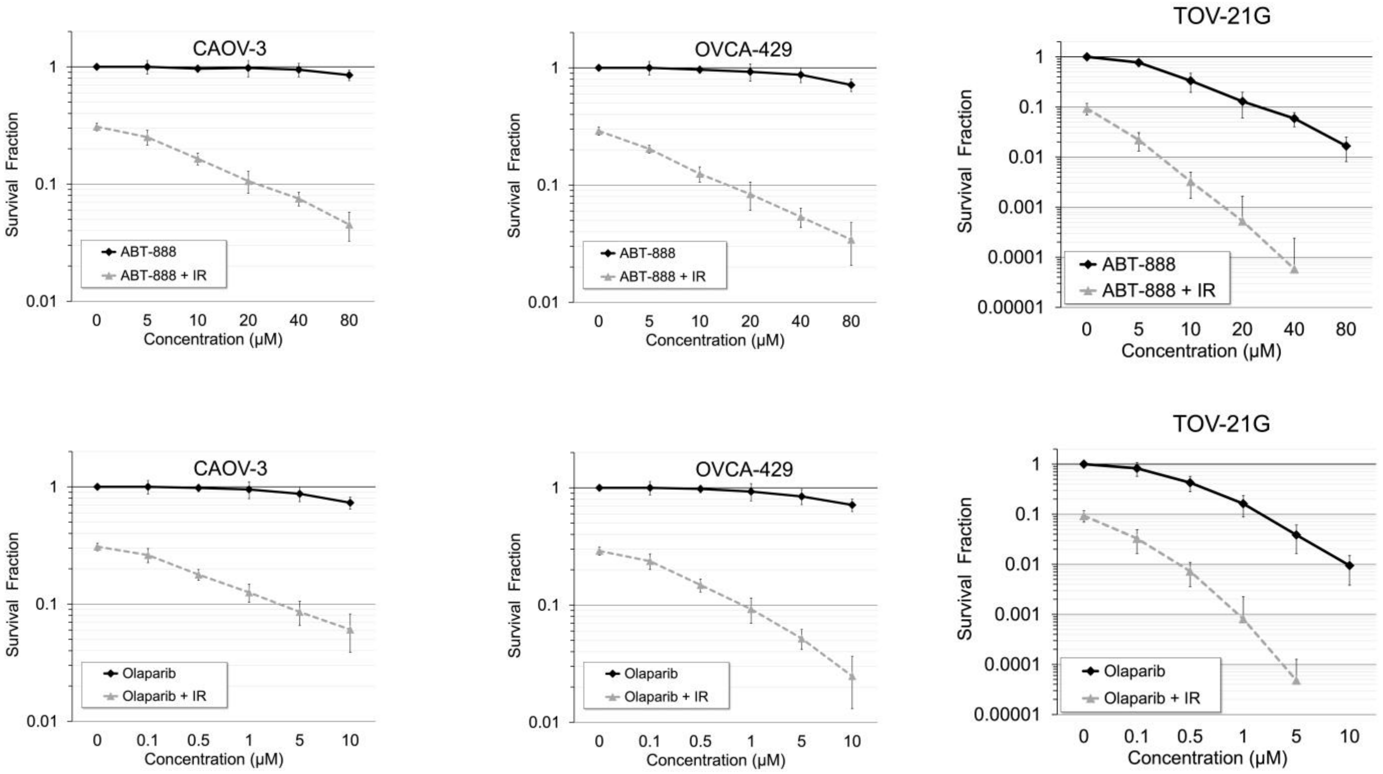

Figure S1. Dose optimization for ABT-888 and Olaparib. Clonogenic analysis of CAOV-3, OVCA-429, and TOV-21G cell lines treated with different concentrations of ABT-888 or Olaparib for 24 h with or without 4 Gy of ionizing radiation. Cells incubated with the same amount of a vehicle (DMSO) were used as a control. Results of clonogenic analysis were normalized to non-treated control and presented as the mean ±SD for quadruplicate samples.

The optimal doses of ABT-888, Olaparib, LY294002, and MK-2206 have been determined in preliminary studies (Figure S1) and are consistent with the most commonly used in a large number of published studies (1-7).

References:

1. Marzio A, Puccini J, Kwon Y, Maverakis NK, Arbini A, Sung P, et al. The F-Box Domain-Dependent Activity of EMI1 Regulates PARPi Sensitivity in Triple-Negative Breast Cancers. Mol Cell. 2019;73(2):224-37 e6.
2. Neijenhuis S, Bajrami I, Miller R, Lord CJ, Ashworth A. Identification of miRNA modulators to PARP inhibitor response. DNA Repair (Amst). 2013;12(6):394-402.
3. Balvers RK, Lamfers ML, Kloezeman JJ, Kleijn A, Berghauser Pont LM, Dirven CM, et al. ABT-888 enhances cytotoxic effects of temozolomide independent of MGMT status in serum free cultured glioma cells. J Transl Med. 2015;13:74.
4. Jue TR, Nozue K, Lester AJ, Joshi S, Schroder LB, Whittaker SP, et al. Veliparib in combination with radiotherapy for the treatment of MGMT unmethylated glioblastoma. J Transl Med. 2017;15(1):61.
5. Guo Y, Chen Y, Liu LB, Chang KK, Li H, Li MQ, et al. IL-22 in the endometriotic milieu promotes the proliferation of endometrial stromal cells via stimulating the secretion of CCL2 and IL-8. Int J Clin Exp Pathol. 2013;6(10):2011-20.
6. Li M, Wang J, Wang C, Xia L, Xu J, Xie X, et al. Microenvironment remodeled by tumor and stromal cells elevates fibroblast-derived COL1A1 and facilitates ovarian cancer metastasis. Exp Cell Res. 2020;394(1):112153.
7. Tao QS, Huang HL, Chai Y, Luo X, Zhang XL, Jia B, et al. Interleukin-6 up-regulates the expression of interleukin-15 is associated with MAPKs and PI3-K signaling pathways in the human keratinocyte cell line, HaCaT. Mol Biol Rep. 2012;39(4):4201-5.
